# Citral Is Renoprotective for Focal Segmental Glomerulosclerosis by Inhibiting Oxidative Stress and Apoptosis and Activating Nrf2 Pathway in Mice

**DOI:** 10.1371/journal.pone.0074871

**Published:** 2013-09-17

**Authors:** Shun-Min Yang, Kuo-Feng Hua, Yu-Chuan Lin, Ann Chen, Jia-Ming Chang, Louis Kuoping Chao, Chen-Lung Ho, Shuk-Man Ka

**Affiliations:** 1 Graduate Institute of Life Sciences, National Defense Medical Center, Taipei, Taiwan, R.O.C; 2 Department of Pathology, Tri-Service General Hospital, National Defense Medical Center, Taipei, Taiwan, R.O.C; 3 Department of Biotechnology and Animal Science, National Ilan University, Ilan, Taiwan, R.O.C; 4 Department of Pharmacology, Institute for Drug Evaluation Platform, Development Center for Biotechnology, Taipei, Taiwan, R.O.C; 5 Department of Cosmeceutics, China Medical University, Taichung, Taiwan, R.O.C; 6 Division of Wood Cellulose, Taiwan Forestry Research Institute, Taipei, Taiwan, R.O.C; 7 Graduate Institute of Aerospace and Undersea Medicine, National Defense Medical Center, Taipei, Taiwan, R.O.C.; Beth Israel Deaconess Medical Center, Harvard Medical School, United States of America

## Abstract

The pathogenesis of focal segmental glomerulosclerosis (FSGS) is considered to be associated with oxidative stress, mononuclear leukocyte recruitment and infiltration, and matrix production and/or matrix degradation, although the exact etiology and pathogenic pathways remain to be determined. Establishment of a pathogenesis-based therapeutic strategy for the disease is clinically warranted. Citral (3,7-dimethyl-2,6-octadienal), a major active compound in *Litsea* *cubeba* , a traditional Chinese herbal medicine, can inhibit oxidant activity, macrophage and NF-κB activation. In the present study, first, we used a mouse model of FSGS with the features of glomerular epithelial hyperplasia lesions (EPHLs), a key histopathology index of progression of FSGS, peri-glomerular inflammation, and progressive glomerular hyalinosis/sclerosis. When treated with citral for 28 consecutive days at a daily dose of 200 mg/kg of body weight by gavage, the FSGS mice showed greatly reduced EPHLs, glomerular hyalinosis/sclerosis and peri-glomerular mononuclear leukocyte infiltration, suggesting that citral may be renoprotective for FSGS and act by inhibiting oxidative stress and apoptosis and early activating the Nrf2 pathway. Meanwhile, a macrophage model involved in anti-oxidative and anti-inflammatory activities was employed and confirmed the beneficial effects of citral on the FSGS model.

## Introduction

Focal segmental glomerulosclerosis (FSGS) manifests with heavy proteinuria in association with focal, but progressive, glomerular sclerosis in the kidney [1-3]. The frequency of end-stage renal disease in patients with FSGS was found to be as high as 78% in long-term follow-up studies [4,5]. Although corticosteroids and other immunomodulatory agents are commonly used to treat these patients [6,7], they result in an unsatisfactory outcome in terms of progression of renal inflammation and fibrosis [8,9] and have various side-effects [10,11]. In addition, the administration of such agents is mostly based on empirical decisions, rather than on targeting specific pathogenic pathways [12]. The establishment of a pathogenesis-based therapeutic strategy is therefore clinically significant.

Although the etiology and pathogenesis of FSGS are poorly understood, its pathogenic pathways may involve oxidative stress [13-15], inflammation associated with mononuclear leukocyte recruitment [16-18], and promotion of matrix production and/or degradation [19]. Oxidative stress is caused by increased production of reactive oxygen species (ROS), nitric oxide (NO), and/or impaired antioxidant capacity [20,21], leading to necrosis, inflammation, apoptosis, and fibrosis in the kidney [22]. Recent advances in understanding the mechanisms involved in renal fibrosis have shown that the NAD(P)H oxidase enzyme complex in infiltrating leukocytes or intrinsic renal cells is involved in ROS production in renal lesions in a rat chronic renal failure model [22] and mesangial cell injury model [23]. Blockade of oxidative stress can prevent renal sclerosis by inhibiting inflammatory responses and apoptosis [21,24]. In addition, nuclear factor E2-related factor 2 (Nrf2), a transcription factor, decreases oxidative stress in various types of cells and tissues by binding to the antioxidant response element in the promoter region of a number of genes encoding antioxidant and phase 2 enzymes, including heme oxygenase 1 (HO-1), NAD(P) H: quinone oxidoreductase 1 (NQO1), glutathione peroxidase, catalase, and superoxide dismutase [25-27]. In renal conditions, Nrf2 has been shown to regulate cellular production of antioxidants and thus protects against oxidative stress in chronic renal failure [28,29], renal inflammation [30] and fibrosis [31,32].

Citral (3,7-dimethyl-2,6-octadienal), a major active compound in *Litsea* *cubeba* , a traditional Chinese herbal medicine, has been shown to inhibit oxidant activity [33,34] and NO production [35], macrophage activation, NF-κB activation, and cytokine production [36]. It has also been shown to ameliorate animal models of inflammation [37,38]. On the basis of its anti-oxidant and anti-inflammatory effects, we tested the hypothesis that Citral may prevent the development of FSGS lesions in a mouse model of FSGS that features: (1) glomerular epithelial hyperplasia lesions (EPHLs), a key histopathology index of progression of FSGS [39], (2) peri-glomerular inflammation, and (3) progressive glomerular hyalinosis/sclerosis. Our data suggest that Citral may be a potential renoprotective agent for FSGS and act by early inhibiting oxidative stress and activating the Nrf2 pathway.

## Materials and Methods

### Ethics statement

All animal experiments were performed with the ethical approval of the Institutional Animal Care and Use Committee of The National Defense Medical Center, Taiwan and according to the ethical rules in the NIH *Guide for the Care and Use of Laboratory Animals*. The animals were maintained in the Animal Center of the National Defense Medical Center (Taipei, Taiwan).

### Preparation of Citral (3, 7-dimethyl-2-7-octadienal)

Fruits of *Litsea* *cubeba* , a traditional Chinese herbal medicine, were obtained from the Lienhuachih Research Center of the Taiwan Forestry Research Institute, Taiwan, in central Taiwan. One kg of the fruits of *Litsea* *cubeba* was placed in a round-bottom flask to which 3 liter of distilled water was added and the mixture refluxed for 8 h. The essential oil layer above the water was separated, dried with anhydrous sodium sulfate, and placed in specimen bottles. Five grams of fresh oil was dissolved in 5 ml of a 1:8 mixture of ether / *n*-hexane and purified by HPLC on a Phenomenex, Luna Silica (2) column (25 cm long, 1 cm i.d., 5.0 µm) using a Smartline RI Detector 2400 and a Knauer 1000 pump (both from Knauer, Berlin, Germany). The separation conditions were as follows: 1 ml was injected for each separation, the flow rate was 4 ml/min, and the mobile phase was a 1:8 mixture of ether/n-hexane. Citral, 3, 7-dimethyl-2-7-octadienal, was eluted with a retention time of 6.08 min. Its structure was confirmed by comparison of the physical and spectral data (including optical rotation EI-MS, ^13^C-NMR, and ^1^H-NMR) with previously reported values [40]. Nuclear magnetic resonance spectra were recorded on a Bruker Avance 400MHz FT-NMR spectrometer. Mass spectra were obtained using a Finnigan MAT-95S mass spectrometer.

### Mouse FSGS model and experimental protocol

A progressive type of mouse FSGS model was used, particularly characterized by EPHLs, a key histopathology index of progression of FSGS, peri-glomerular inflammation, and progressive glomerular hyalinosis/sclerosis [15,39]. The FSGS model was induced in 8-week-old male BALB/c mice (National Laboratory Animal Center, Taipei, Taiwan) by intravenous injection of a single dose of adriamycin (0.10 mg/10 g body weight) (Pfizer, New York, NY) as described previously [15]. Starting three days before adriamycin injection (recorded as day 0 for FSGS model induction), groups of mice (n = 7 each) were given a daily dose of Citral (200 mg/kg of body weight) or vehicle (corn oil) by gavage, and were sacrificed on day 7, 14, or 28 after FSGS model induction. Age- and sex-matched BALB/c mice were used as normal controls. Urine samples were collected in metabolic cages on days 3, 7, 14, 21, and 28. Renal cortical tissues and blood samples were collected when the mice were sacrificed and stored appropriately for further analysis. The concentration of urine protein was determined using BCA kits (Pierce, Rockford, IL) as described previously [41] and normalized to urine creatinine (Cr) levels measured using kits (Wako Pure Chemical Industries, Osaka, Japan), as described previously [15]. Serum levels of blood urea nitrogen (BUN) and Cr were determined using BUN kits and Cr kits (both from Fuji, Dry-Chem Slide, Fuji Film Medical, Tokyo, Japan), as described previously [42].

### Pathologic evaluation

Renal tissues were formalin-fixed, embedded in paraffin, and sections (4 µm) prepared and stained with hematoxylin and eosin (H&E) for renal histopathology as described previously [15] or TUNEL stained for apoptosis as described previously [43]. Renal pathology was examined and renal lesions scored as described previously [39]. For EPHLs and sclerosis, at least 50 glomeruli in sections were examined for each mouse. To detect apoptosis in renal tissues, TUNEL staining was performed using an *in situ* apoptosis detection kit (Chemicon, Temecula, CA) according to the manufacturer’s instructions. For immunohistochemistry (IHC), methyl Carnoy’s solution fixed and paraffin-embedded renal sections were prepared and incubated with goat antibodies against mouse collagen IV (Col-IV) (Southern Biotech, AL) or rabbit antibodies against desmin (Lab Vision, Fremont, CA), phosphorylated mouse NF-κB p65 (Cell Signaling Technology, MA), F4/80 (Serotec, Raleigh, NC), or CD3 (Serotec), then with horseradish peroxidase (HRP)-conjugated rabbit anti-goat IgG or swine anti-rabbit IgG antibodies (both from Dako, Carpinteria, CA). Quantitative image analysis software (Pax-it; Paxcam, Villa Park, IL) was used to score Col-IV staining and the number of phosphorylated NF-κB p65-, CD3-, F4/80-, or TUNEL-positive cells as described previously [42].

### Measurement of superoxide anion and NO

Superoxide anion levels in serum, urine, and kidney tissues were determined as described previously [44]. The results were expressed as relative luminescence units (RLU) per 15 min per milliliter (i.e., RLU/15 min/ml) for serum and urine samples or per milligram dry weight (i.e., RLU/15 min/mg dry weight) for kidney tissues. In addition, renal superoxide anion levels were measured by dihydroethidium (DHE) binding, fluorescence being quantified by counting the percentage of the total nuclei that were positive per kidney cross section as described previously [44]. NO levels in serum and urine were measured using NO detection kits (Abcam, Cambridge, MA) according to the manufacturer’s instructions.

### Measurement of renal Nrf2, NAD(P) H subunit p47^phox^ (p47^phox^), NQO1, HO-1, caspase-3, caspase-8, caspase-9, Bcl-2, and Bax

Cytoplasmic and nuclear proteins from renal tissues were prepared using a kit (Active Motif, Tokyo, Japan) according to the manufacturer’s instructions and target proteins detected by immunoblotting using goat antibodies against mouse Nrf2 or p47^phox^ (Santa Cruz Biotechnology, Santa Cruz, CA) or rabbit antibodies against mouse NQO1 (Abcam), caspase-3, caspase-8, or caspase-9 (all from Cell Signaling Technology, MA) or Bcl-2 or Bax (both from Santa Cruz Biotechnology), then with HRP-conjugated rabbit anti-goat IgG antibodies or goat anti-rabbit IgG antibodies (both from Santa Cruz Biotechnology) as described previously [45]. Anti-β-actin antibody (Santa Cruz Biotechnology) was used as internal controls for the nuclear and cytosolic target proteins, respectively. Renal HO-1 levels were measured using a commercial ELISA kit (R&D Systems, Minneapolis, MN) according to the manufacturer’s instructions.

### *In vitro* experiments with macrophages

RAW-BlueTM cells (murine macrophages RAW264.7 stably transfected with the NF-κB reporter gene) purchased from InvivoGen (San Diego, CA). LPS (from *Escherichia coli* 0111:B4) and mouse antibodies against phospho-ERK1/2, phospho-JNK1/2, and phospho-p38 (all from Sigma, St. Louis, MO). Rabbit antibodies against mouse ERK1, JNK1, and p38, and HRP-labeled second antibodies were purchased from Santa Cruz Biotechnology, and IL-1β, IL-6, and TNF-α ELISA kits from R&D Systems. ROS production assay, NO production assay, NF-κB reporter assay, ELISA, and Western blotting were performed as described previously [46].

### Statistical analysis

The results for animal model are presented as the mean ± SEM. Comparison between two groups was performed using Student’s *t* test. For *in vitro* experiments, all values are given as mean ± SD. Data analysis involved one-way ANOVA with a subsequent Scheffe´ test. A value of *p* < 0.05 was considered statistically significant.

## Results

### Citral ameliorated mouse FSGS model

#### Proteinuria, renal function, and renal pathology

**Figure 1:**
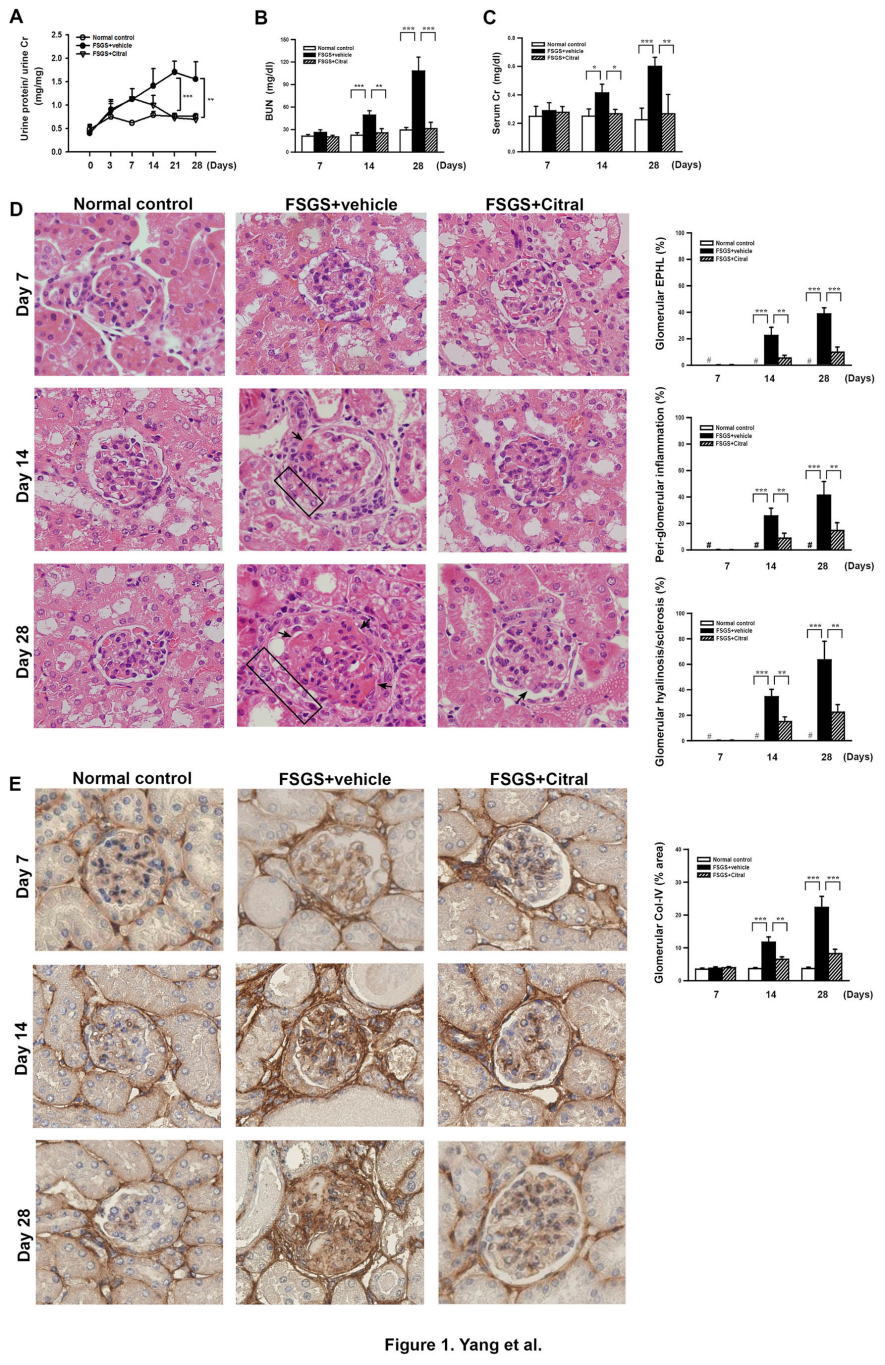
Urine protein, renal function, renal histopathology, and hyalinosis/sclerosis. (A) Urine protein time-course study. (B) Serum BUN levels on days 7, 14, and 28. (C) Serum creatinine levels on days 7, 14, and 28. (D) Kidney histopathological evaluation by H&E staining on days 7, 14, and 28. The arrows indicate hyalinosis/sclerosis, and the rectangles EPHLs. (E) Immunohistochemical staining for renal Col-IV. In D and E, the original magnification was 400× and the scoring is shown on the right. In the histograms, the data are the mean±SEM for seven mice per group. *p < 0.05, **p < 0.01, ***p < 0.005. #, not detectable.

As shown in Figure 1A, compared to normal control mice, disease-control FSGS mice treated with vehicle (FSGS+vehicle mice) showed significantly increased proteinuria compared to normal control mice at day 7 after disease induction up to day 28 when the mice were sacrificed. However, in FSGS+Citral mice, this effect was slightly inhibited at day 14 and markedly inhibited at days 21 (*p* < 0.005) and 28 (*p* < 0.01) compared to FSGS+vehicle mice. In renal function assessment, significantly higher serum levels of BUN (Figure 1B) and Cr (Figure 1C) were seen in FSGS+vehicle mice at days 14 (both *p* < 0.05) and 28 (both *p* < 0.005) than in normal control mice and these effects were almost, or completely, suppressed in FSGS+Citral mice (all *p* < 0.05) (Figure 1B and 1C). Light microscopy showed that characteristic glomerular EPHLs, suggestive of progression of FSGS lesions, glomerular hyalinosis/sclerosis and peri-glomerular inflammation were both seen at days 14 and 28 in FSGS+vehicle mice, but these renal lesions were greatly decreased in FSGS+Citral mice (all *p* < 0.01) (Figure 1D). Furthermore, FSGS+vehicle mice showed strong renal Col-IV expression at days 14 and 28 and this was markedly inhibited in FSGS+Citral mice (all *p* < 0.01) (Figure 1E).

#### Oxidative stress in renal tissue, serum, or urine

**Figure 2:**
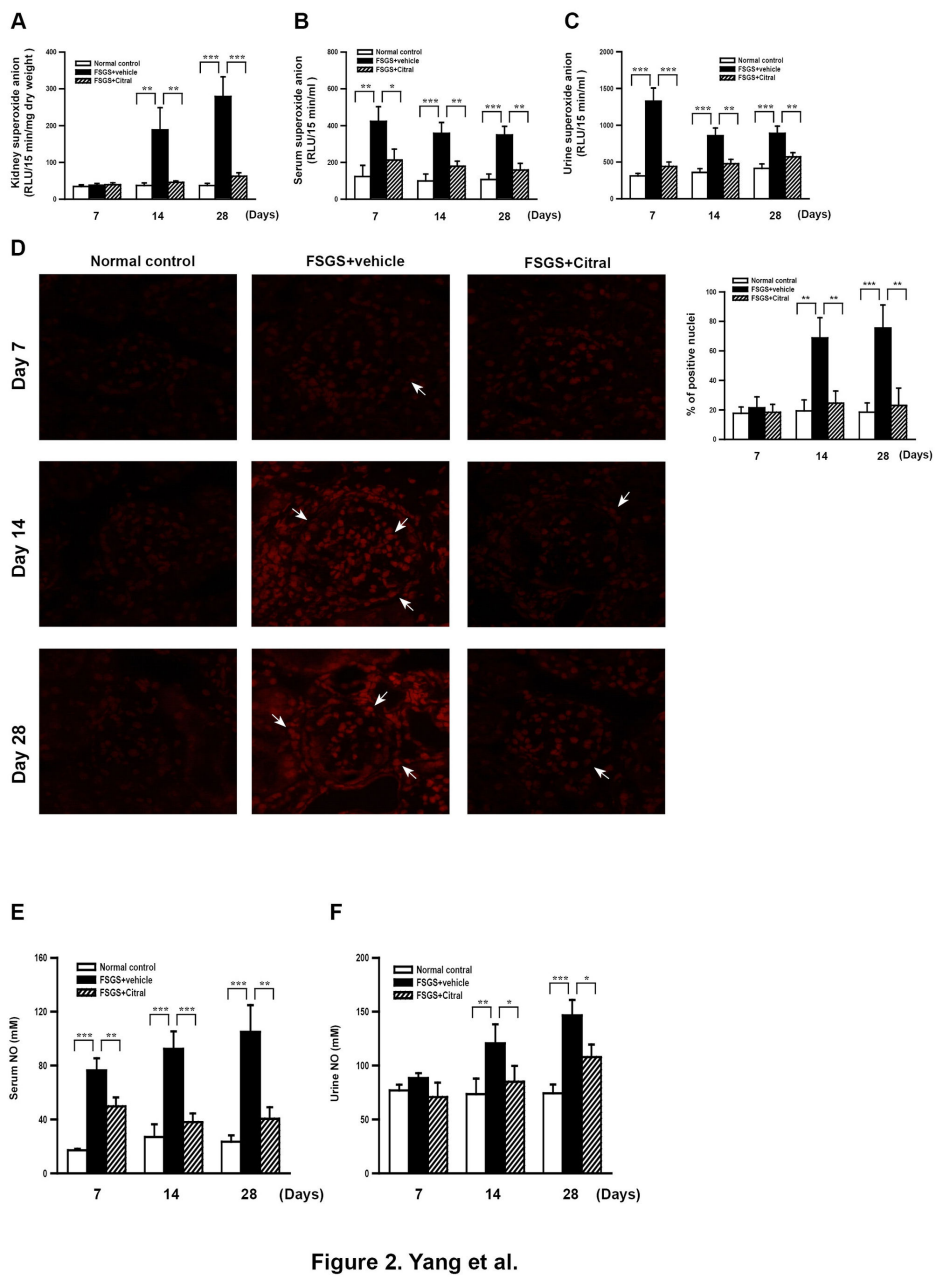
ROS/NO levels. (A-C) Superoxide anion levels in renal tissue (A), serum (B), and urine (C). (D) Kidney in situ ROS production demonstrated by DHE labeling. The arrows indicate positive staining cells. Original magnification, 400×. The scoring is shown on the right. (E, F) NO levels in serum (E) and urine (F). In the histograms, the data are the mean±SEM for seven mice per group. *p < 0.05, **p < 0.01, ***p < 0.005.

As shown in Figure 2A-C, compared to normal control mice, FSGS+vehicle mice had significantly higher levels of superoxide anion in renal tissues, serum, and urine (all *p* < 0.01) at days 14 and 28 and levels were markedly decreased in FSGS+Citral mice compared to FSGS+vehicle mice (all *p* < 0.01). When ROS levels in renal tissues were examined by detection of DHE levels, FSGS mice showed significantly increased renal DHE levels compared to normal control mice at days 14 and 28 (both *p* < 0.01) and this effect was suppressed in FSGS+Citral mice (both *p* < 0.01) (Figure 2D). In addition, FSGS+vehicle mice showed significantly increased NO levels in serum (Figure 2E) and urine (Figure 2F) compared to normal control mice at days 14 (both *p* < 0.01) and 28 (both *p* < 0.005) and levels were significantly reduced in FSGS+Citral mice compared to FSGS+vehicle at days 14 and 28 (all *p* < 0.05).

**Figure 3:**
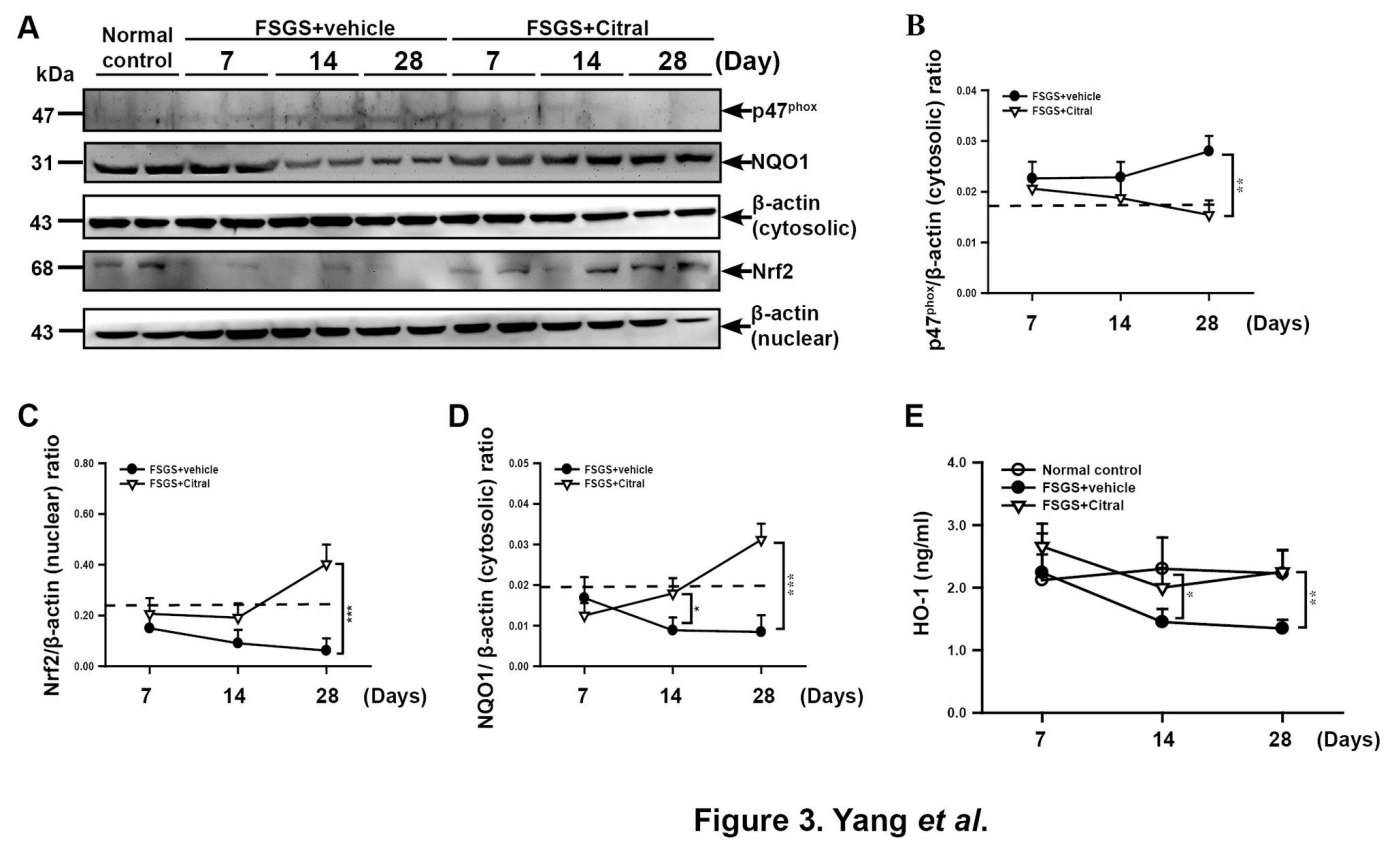
Renal nuclear Nrf2 levels, cytosolic p47^phox^, NQO1 and HO-1 levels. (A) Representative Western blot showing levels of cytosolic p47^phox^ and NQO1 and nuclear Nrf2 in kidney tissues. (B-D) Quantification of the p47 ^phox^/β-actin (cytosolic) ratio (B), the Nrf2/β-actin (nuclear) ratio (C), and the NQO1/β-actin (cytosolic) ratio (D). The horizontal dashed lines indicate levels in normal control mice. (E) HO-1 levels in the kidney. In B-E, the data are the mean±SEM for seven mice per group. *p < 0.05, **p < 0.01, ***p < 0.005.

Since the antioxidant signaling pathway can be activated by reduced production of NAD(P)H oxidase or by activation of Nrf2, we measured protein levels of p47^phox^, nuclear Nrf2 (activation), and HO-1 in the kidney to evaluate the effects of Citral on this pathway. As shown in Figure 3A and 3B, p47 ^phox^ protein levels were significantly increased in FSGS+vehicle at day 28 (*p* < 0.01) and this effect was inhibited by Citral administration (*p* < 0.01). As shown in Figure 3A and 3C, the nuclear Nrf2 expression was significantly decreased in FSGS+vehicle at days 14 and 28 (both *p* < 0.05) compared to normal control mice. This effect was inhibited at day 14 in FSGS+Citral mice, although not statistically significant, and obviously, the FSGS+Citral mice showed a greatly increased nuclear Nrf2 levels at day 28 compared to FSGS+vehicle (*p* < 0.005) and those of normal control mice (*p* < 0.05). In addition, cytosolic levels of NQO1 (Figure 3A and D) in FSGS+vehicle mice was significantly decreased at days 14 and 28 compared to those of normal control mice (both *p* < 0.05), whereas levels in Citral-treated mice were greatly restored to much closer at day 14 or significantly higher at day 28 (*p* < 0.01) than those observed in normal control mice. Moreover, compared to FSGS+vehicle mice with greatly reduced cytosolic levels of HO-1 at days 14 (p < 0.05) and 28 (p < 0.01), respectively, compared to normal control mice (Figure 3E), FSGS+Citral mice showed a significantly elevated HO-1 levels at the both points (*p* < 0.05 or *p* < 0.01).

#### Podocyte injury, apoptosis, caspases, Bax/Bcl-2 ratio in renal tissue

**Figure 4:**
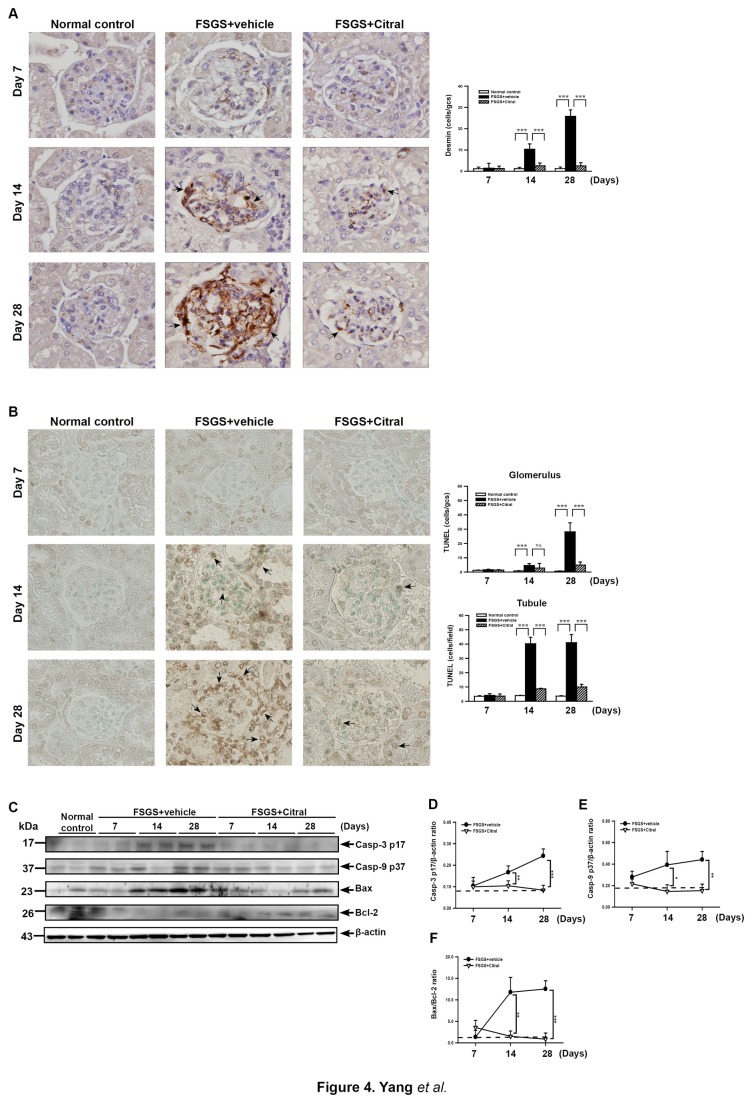
Podocyte injury and renal apoptosis in the glomerulus and tubule. (A) Podocyte injury detected in glomeruli by immunohistochemical staining for desmin. The black arrows indicate podocytes. Original magnification, 400×. Scoring of desmin expression in renal tissue is shown on the right. (B) TUNEL staining in renal tissues at day 7, 14, and 28. Original magnification, 400×. The arrows indicate positively stained cells. The scoring is shown on the right. (C) Representative Western blot for the active forms of caspase-3, caspase-9, Bax and Bcl-2, with β-actin as the internal control. (D-F) Active caspase-3/β-actin ratio (D), active caspase-9/β-actin ratio (E), and Bax/Bcl-2 ratio (F). In the histograms, the data are the mean±SEM for seven mice per group. *p < 0.05, **p < 0.01, ***p < 0.005.

The protective effect of Citral on podocytes was evaluated by detecting the glomerular expression of desmin, a marker of podocyte injury, using IHC. As shown in Figure 4, FSGS+vehicle mice showed significantly increased number of podocytes that expressed desmin at days 14 and 28, compared to normal control mice (both *p* < 0.005), but this effect was greatly inhibited in FSGS+Citral mice (*p* < 0.005) (Figure 4A). Furthermore, as shown by TUNEL staining (Figure 4B), although the FSGS+vehicle mice showed markedly increased renal apoptosis levels in the glomerulus and tubular epithelial cells at days 14 and 28 compared to normal control mice (both *p* < 0.005), this effect was significantly inhibited in FSGS+Citral mice at day 28 in the glomerulus (*p* < 0.005) and both points in the renal tubule. When renal levels of activated caspase-3, caspase-8, and caspase-9 were measured, levels of the mature form (p17 fragment) of caspase-3 were greatly increased in FSGS+vehicle mice compared to normal control mice on days 14 and 28 (both *p* < 0.01) and this effect was significantly inhibited in FSGS+Citral mice at days 14 and 28 (both *p* < 0.01) (Figure 4C and 4D). In addition, as shown in Figure 4C and 4E, levels of the mature form (p37 fragment) of caspase-9 were greatly increased in FSGS+vehicle mice (*p* < 0.01) and this effect was suppressed in FSGS+Citral mice at days 14 and 28 (both *p* < 0.05). There was no detectable difference in levels of the mature form (p18 fragment) of renal caspase-8 between FSGS+vehicle mice, FSGS+Citral mice, and normal control mice (data not shown). Since an increased Bax/Bcl-2 ratio is associated with caspase-9 and caspase-3 activation [45], we then measured renal levels of Bax and Bcl-2 and found that the Bax/Bcl-2 ratio was increased in FSGS+vehicle mice compared to normal control mice at days 14 and 28 (both *p* < 0.01) and this effect was significantly decreased in FSGS+Citral mice at days 14 and 28 (both *p* <0.01) (Figure 4C and 4F). These findings suggest that the reduction in renal apoptosis seen in FSGS+Citral mice is due to suppression of the intrinsic pathway of apoptosis.

#### NF-κB activation and MCP-1 expression in renal tissues

**Figure 5:**
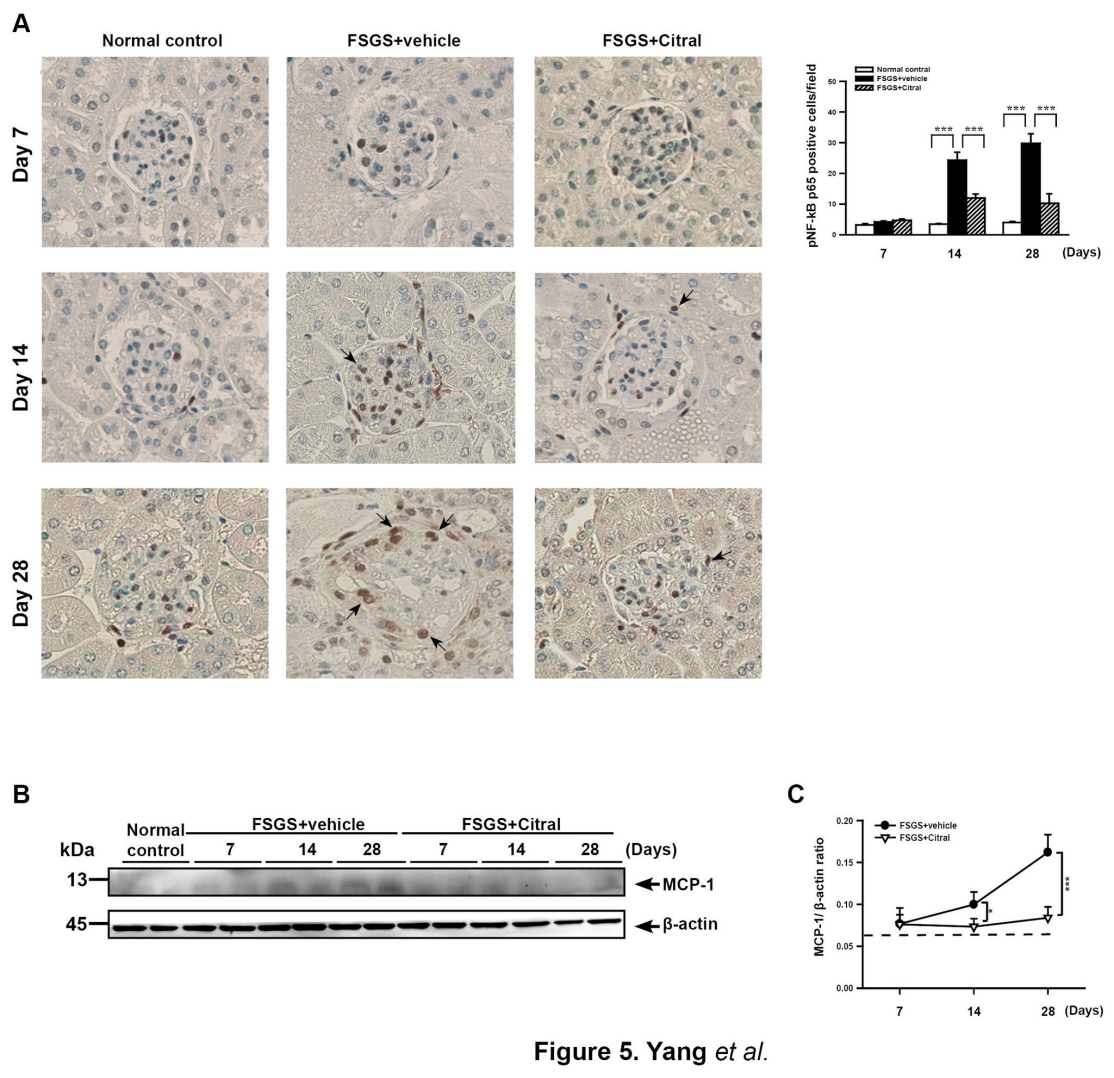
Renal NF-κB activation and MCP-1 expression. (A) Detection of NF-κB p65 by immunohistochemical staining. Original magnification, 400×. The arrows indicate positively stained cells. The scoring is shown on the right. (B) Western blot of MCP-1 levels in renal tissues, with β-actin as the internal control for cytosolic protein. (C) MCP-1/β-actin ratio. In the histograms, the data are the mean±SEM for seven mice per group. *p<0.05, ***p<0.005.

NF-κB activation and the subsequent induction of expression of various proteins, such as MCP-1, are implicated in the development of FSGS [47-49]. As shown in Figure 5A, at days 14 and 28 after disease induction, the FSGS+vehicle mice showed significantly increased renal nuclear translocation of phosphorylated NF-κB p65 compared to normal control mice (both *p* < 0.005) and this effect was significantly inhibited in the FSGS+Citral mice (both *p* < 0.005). In addition, FSGS+vehicle mice had significantly higher renal MCP-1 levels than normal control mice at days 14 and 28 (both *p* <0.05) and this effect was significantly inhibited in FSGS+Citral mice at days 14 and 28 (both *p* < 0.05) (Figure 5B and 5C).

#### Renal infiltration of T cells and macrophages

**Figure 6:**
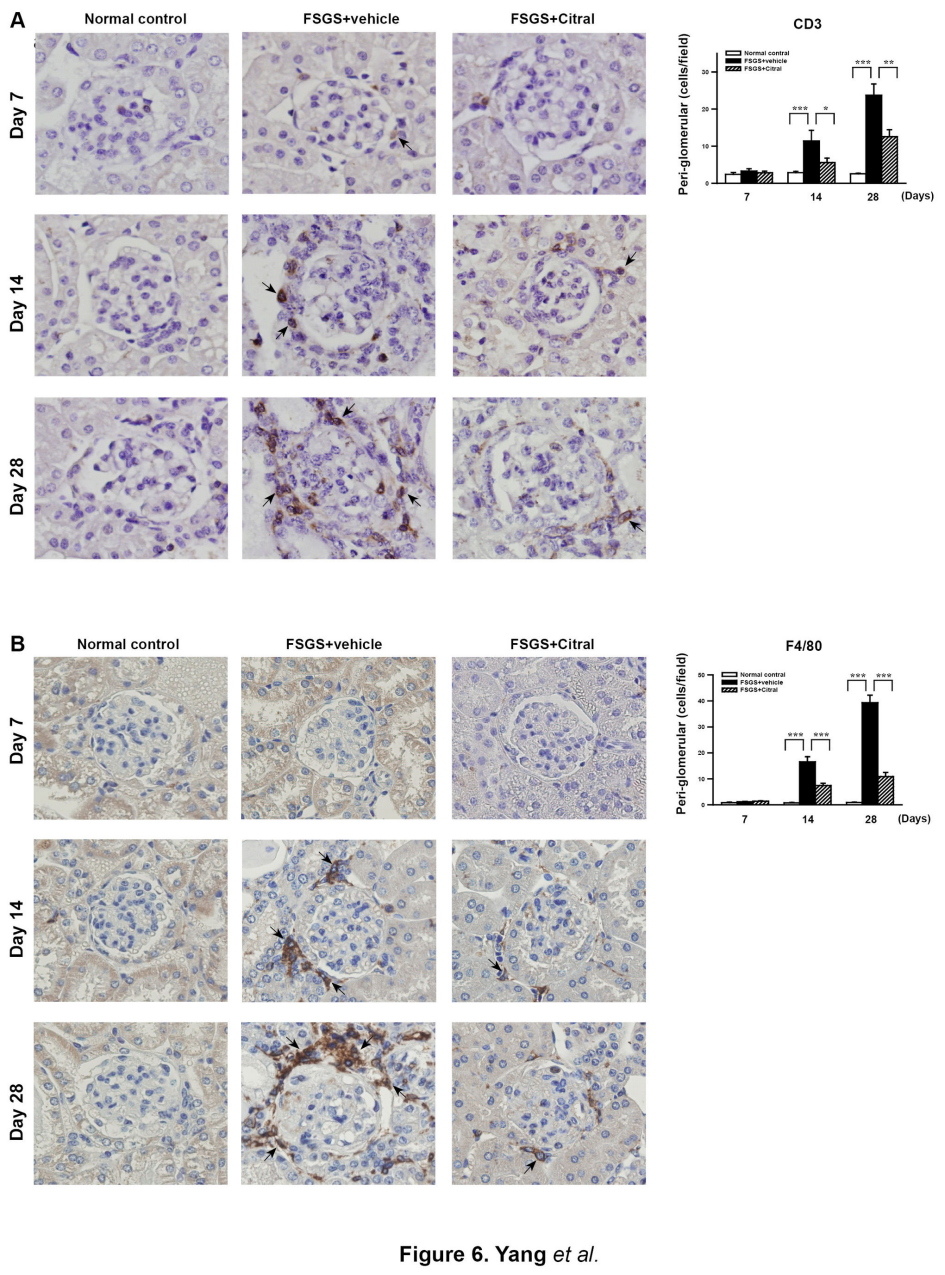
Renal T cell and macrophage infiltration. Detection of (A) CD3^+^ T cells or (B) F4/80^+^ monocytes/macrophages by immunohistochemical staining. The arrows indicate positively stained cells. Original magnification, 400×. The scoring is shown on the right. The data are the mean±SEM for seven mice per group. *p < 0.05, **p < 0.01, ***p < 0.005.

Renal mononuclear leukocyte infiltration is seen in renal tissues of FSGS mice [16,17,50-52]. As shown in Figure 6A, although significantly increased renal peri-glomerular infiltration of T cells (CD3^+^) was seen at days 14 and 28 in FSGS+vehicle mice compared to normal control mice (both *p* < 0.005), this effect was markedly inhibited in FSGS+Citral mice (both *p* < 0.05). Similarly, as shown in Figure 6B, FSGS+vehicle mice showing significantly increased peri-glomerular infiltration of macrophages (F4/80^+^) compared to normal control mice at days 14 and 28 (both *p* < 0.005) and this effect was also significantly decreased in FSGS+Citral mice (both *p* < 0.005).

### Citral inhibited oxidative stress and inflammatory activities of macrophages

#### ROS/NO, NF-κB and pro-inflammatory cytokines

**Figure 7:**
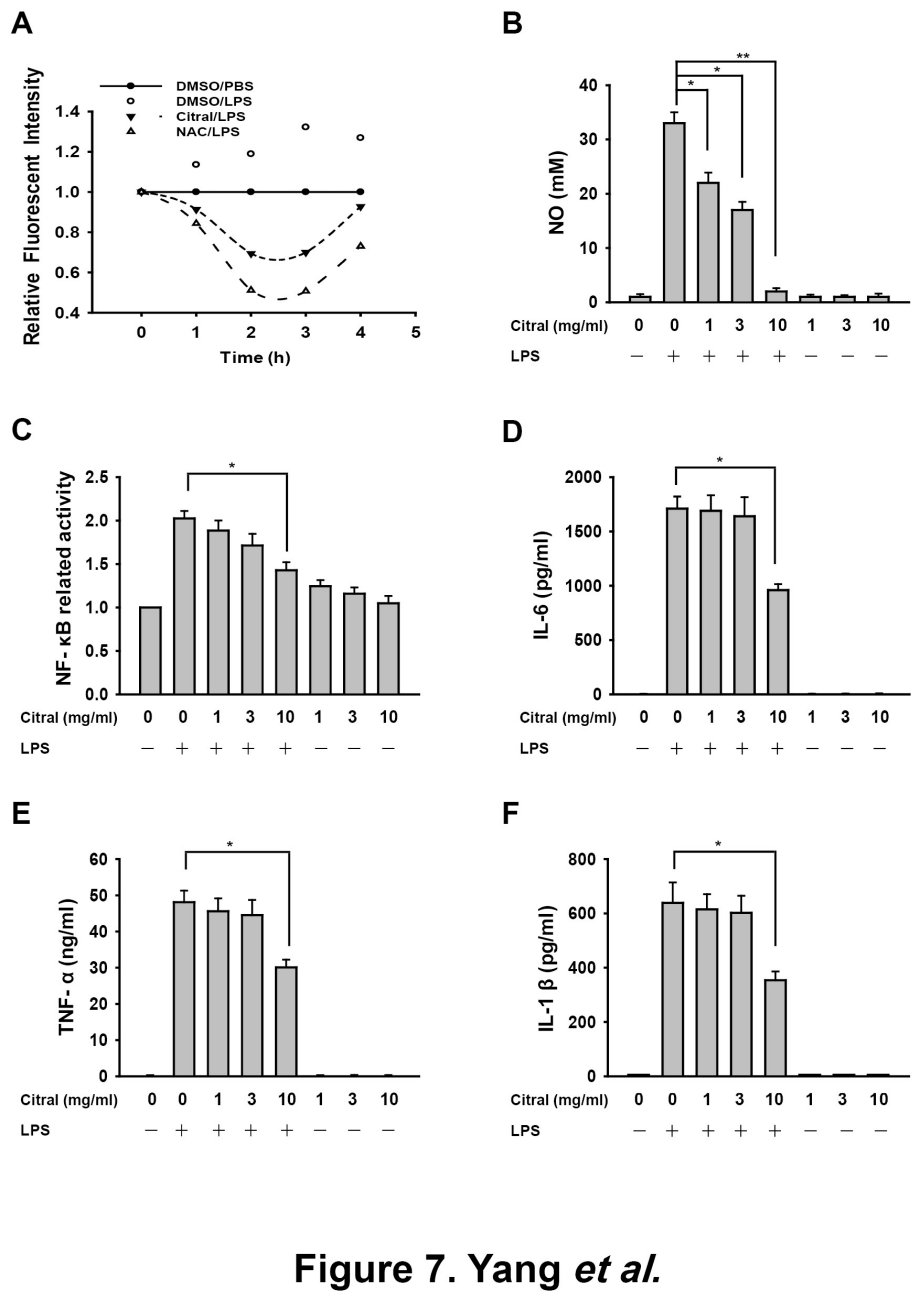
*In vitro* ROS generation and inflammatory mediator expression. (A) RAW 264.7 macrophages were incubated for 30 min with or without 10 µg/ml Citral or 10 mM N-acetyl cysteine (NAC), then for 0-4 h with or without addition of 1 µg/ml of LPS. ROS production was measured as the relative fluorescence intensity. (B) RAW 264.7 macrophages were incubated for 30 min with or without the indicated concentrations of Citral, then for 24 h with or without addition of 1 µg/ml of LPS, then NO generation in the culture medium was measured by the Griess reaction. (C) RAW-Blue^TM^ cells were incubated for 30 min with or without the indicated concentration of Citral, then for 24 h with or without addition of 1 µg/ml of LPS, then secreted embryonic alkaline phosphatase activity was measured using QUANTI-Blue^TM^. (D-F) RAW 264.7 macrophages were incubated for 30 min with or without the Citral, then for 24 h with or without addition of 1 µg/ml of LPS, then IL-6 (D), TNF-α (E), and IL-1β (F) in the culture medium were measured by ELISA. The data are expressed as the mean ± SD for three separate experiments. *p < 0.05, **p < 0.01 compared to the LPS-treated group.

The anti-oxidative and anti-inflammatory activities of Citral were examined using LPS-activated RAW 264.7 macrophages. The LPS-induced increase in ROS production was reduced by incubation with Citral (10 µg/ml) or NAC (10 mM), a potent antioxidant, 30 min before and during LPS stimulation (Figure 7A). The LPS-induced increase in NO generation was inhibited by Citral (*p* < 0.05) (Figure 7B). Further, we examined the effect of Citral on LPS-induced NF-κB activation using NF-κB-dependent alkaline phosphatase reporter cells (RAW-Blue^TM^ cells) and, as shown in Figure 7C, showing that NF-κB transcriptional activity in LPS-stimulated macrophages was reduced by Citral. Furthermore, in the same system, we showed that the secretion levels of IL-6 (Figure 7D), TNF-α (Figure 7E), and IL-1β (Figure 7F) were inhibited by Citral. These data suggest that Citral was anti-oxidative and anti-inflammatory in the LPS-activated macrophages.

#### Phosphorylation of ERK1/2, JNK1/2 and p38

**Figure 8:**
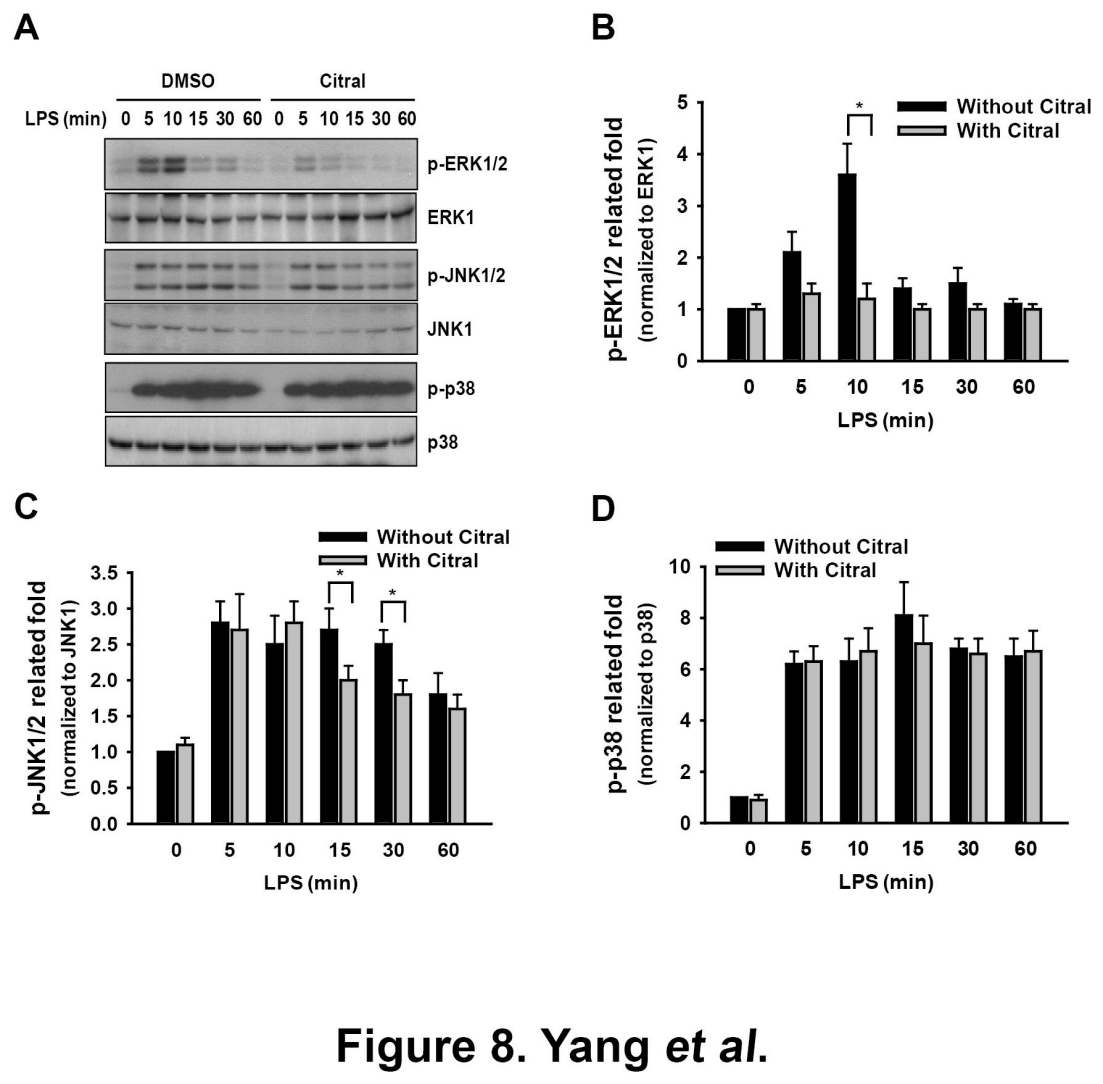
*In vitro* MAPK phosphorylation. (A) RAW 264.7 macrophages were incubated for 30 min with or without 10 µg/ml Citral, then for 0-60 min with or without addition of 1 µg/ml of LPS, then the phosphorylation levels of ERK1/2, JNK1/2, and p38 were measured by Western blotting. In B-D, the results in the phosphorylation levels of ERK1/2 (B), JNK1/2 (C), and p38 (D) are representative of those obtained in three separate experiments and the histogram shows the results for all three experiments expressed as the mean ± SD. *p < 0.05 compared to the corresponding group without Citral.

LPS can induce macrophage activation and the production of pro-inflammatory cytokines by the activation of various signaling pathways, including the mitogen-activated protein kinase (MAPK) signaling pathways [53]. To examine whether the inhibitory effects of Citral on LPS-induced activation of macrophages are associated with MAPK signaling cascades, RAW 264.7 macrophages were treated with LPS in the presence or absence of Citral (10 µg/ml). The results show that although LPS induced increase in the phosphorylation levels of MAPK, including ERK1/2, JNK1/2 and p38 (Figure 8A), this effect was significantly inhibited by Citral for ERK1/2 (*p* < 0.05) (Figure 8B) and JNK1/2 (*p* < 0.05) (Figure 8C), except p38 (Figure 8D). These results suggest that Citral modulated the activation of the MAPK signaling cascades in the LPS-activated macrophages.

## Discussion

Our study demonstrated that Citral, a purified major active component of *Litsea* *cubeba* , had renoprotective effects in a FSGS mouse model, including preventing the kidney from glomerular EPHLs, a key histopathology index of progression of FSGS, and from glomerular hyalinosis/sclerosis and mononuclear leukocyte infiltration. These effects were closely associated with reduced oxidative stress, apoptosis and activation of the Nrf2 pathway before the progression of the FSGS model.

First, we showed that Citral administration inhibited the increase in ROS and NO production and p47^phox^ levels seen in FSGS mice and activated the Nrf2 signaling pathway involving increasing expression of its downstream molecules NQO1 and HO-1 during the early developmental stage of this FSGS model, thus contributing to the beneficial effects of Citral on the treated mice (Figure 3). In this regard, a reduction in nuclear Nrf2 levels is seen in experimental chronic renal failure models [22,54], and impairment of Nrf2 activity is involved in the pathogenesis of oxidative stress- and inflammation-mediated chronic kidney disease [30]. In addition, the NF-κB-mediated inflammatory response is more intense in Nrf2-deficient mice than in wild-type mice [55], and upregulation of Nrf2 suppresses NF-κB activation [56]. Our previous study showed that increased nuclear translocation of Nrf2 is beneficial in the FSGS model [15], confirming previous observations that activation of the Nrf2 signaling pathway is beneficial in experimental chronic renal failure models [22] and in patients with chronic renal insufficiency or failure [28]. Collectively, these findings provide support for a potential therapeutic effect of Citral in renal fibrosis/sclerosis caused by activation of the Nrf2 signaling pathway. However, recently studies using a highly selective Nrf2 agonist bardoxolone suggest that bardoxolone actually worsened proteinuria [57,58], although bardoxolone and related synthetic triterpenoid analogs have been shown to have beneﬁcial effects on prevention and therapy of tissue injury mediated by inflammatory and oxidative stress [59,60]. Further investigation on this discrepancy and related mechanistic pathways involved is warranted.

Second, oxidative stress and inflammation are common features of chronic kidney disease [30,61] and play a critical role in the development of renal fibrosis [31,62]. In this regard, our data showed that Citral was anti-oxidative and anti-inflammatory in a model of activated macrophages (Figures 7, 8). Oxidative stress and inflammation are closely linked in a vicious cycle, as each amplifies the other [63], and oxidative stress can induce inflammation by activating NF-κB and the subsequent production of proinflammatory cytokines [30,64,65], leading to leukocyte activation and the production and release of ROS/NO [66-68]. In the present study, we demonstrated that Citral administration significantly decreased renal NF-κB activation and MCP-1 expression, resulting in significant inhibition of T cell and macrophage infiltration into the kidney in FSGS+Citral mice, and this effect may contribute to the decrease in glomerular EPHLs which can be promoted by these inflammatory cells [39]. Our data also confirm that EPHLs can be used as a reliable tissue marker for monitoring the progression of FSGS. In our previous study, we found that FSGS mice show increased expression of renal TGF-β1 protein [15], and, in the present study, we demonstrated an increase in levels of its downstream protein Col-IV in the kidney, again supporting the idea that Citral protects the kidney from renal fibrosis in FSGS mice by blocking the TGF-β1-dependent fibrosis pathway (Figure 1E).

Podocyte injury has been involved in the pathogenesis of FSGS [69]. We demonstrated protective effect of Citral on podocytes in FSGS+Citral mice. Besides, a number of pathological lesions can arise from oxidative stress-mediated apoptosis [70]. Apoptosis is involved in the development and progression of FSGS [71,72], and, in agreement with this, we showed that inhibition of apoptosis in the kidney by Citral administration was associated with only slight histopathological renal lesions. Furthermore, Citral administration resulted in decreased renal levels of activated caspase-3 and caspase-9 (but not of activated caspase-8) as well as Bax/Bcl-2 ratio (Figure 4C-F). Nrf2-induced expression of the anti-apoptotic protein Bcl-2 has been shown to enhance cell survival [73,74]. Together, these results suggest that inhibition of apoptosis pathway in the kidney may also contribute to the beneficial effects of treatment on FSGS. However, to our knowledge, there have been no reports of an anti-apoptotic effect of Citral on such an inflammation-associated condition in the kidney, and further investigations are needed.

In summary, our results suggest that Citral may have renoprotective potential for renal inflammation and fibrosis in FSGS, based on its anti-oxidant, anti-apoptotic and anti-inflammatory effects. Further studies on its systemic side effects are warranted before it can be considered for a preclinical validation.
